# Risk factors for *Pseudomonas aeruginosa* colonization in non-cystic fibrosis bronchiectasis and clinical implications

**DOI:** 10.1186/s12931-021-01729-5

**Published:** 2021-04-28

**Authors:** Wang Chun Kwok, James Chung Man Ho, Terence Chi Chun Tam, Mary Sau Man Ip, David Chi Leung Lam

**Affiliations:** Department of Medicine, Queen Mary Hospital, University of Hong Kong, 102 Pokfulam Road, Pokfulam, Hong Kong Special Administrative Region China

**Keywords:** Bronchiectasis, *Pseudomonas aeruginosa*, Colonization, Bronchiectasis exacerbation, Non-cystic fibrosis bronchiectasis

## Abstract

**Background:**

*Pseudomonas aeruginosa* is one of the commonest bacteria colonizing the airway in patients with non-cystic fibrosis bronchiectasis. *Pseudomonas aeruginosa* colonization is associated with poor outcomes in patients with bronchiectasis, including rapid decline in lung function, exacerbation frequency and hospitalization.

**Methods:**

A cross-sectional study in Queen Mary Hospital, Hong Kong that included 350 Chinese patients with non-cystic fibrosis bronchiectasis to investigate the risk factors for *Pseudomonas aeruginosa* colonization and clinical implications on disease outcomes.

**Discussions:**

*Pseudomonas aeruginosa* colonization was more commonly found in patients with longer duration of bronchiectasis and those on proton pump inhibitors (PPIs) with adjusted ORs of 1.066 (95% CI = 1.036–1.096, *p* < 0.001) and 2.815 (95% CI = 1.307–6.064, *p* = 0.008) respectively. Patients with *Pseudomonas aeruginosa* colonization have more extensive lung involvement and higher risks of exacerbation requiring hospitalization with adjusted ORs of 2.445 (95% CI = 1.283–4.657, *p* = 0.007) and 2.745 (95% CI = 1.012–7.449, *p* = 0.047) respectively. *Pseudomonas aeruginosa* colonization is more common among patients with longer duration of bronchiectasis and those on PPI. *Pseudomonas aeruginosa* colonization is associated with more extensive lung involvement and higher risks of exacerbation requiring hospitalization.

## Introduction

As a common suppurative respiratory disease among Chinese population, non-cystic fibrosis (non-CF) bronchiectasis is characterized by having micro-organism colonization as well as exacerbation. Both bacteria and non-tuberculous mycobacteria (NTM) can commonly colonize the airway in patients with non-CF bronchiectasis.

Among all bacteria, *Pseudomonas aeruginosa* is one of the commonest colonizers in non-CF bronchiectasis. It was estimated around 25% of patients with non-CF bronchiectasis had chronic colonization with *Pseudomonas aeruginosa* [1–4]. *Pseudomonas aeruginosa* colonization was associated with poor outcome in patients with bronchiectasis [5], which encompassed more extensive and severe bronchiectatic changes on imaging [6], rapid decline in lung function [7, 8], increased frequency of exacerbation and hospitalization [3, 6, 9–11]. *Pseudomonas aeruginosa* colonization is also one of the components within the Bronchiectasis Severity Index (BSI) and FACED scores, which predict prognosis and future hospitalization [12, 13]. BSI consists of clinical (hospitalization and exacerbation history, body mass index, MMRC dyspnea score), spirometric (percentage FEV_1_ predicted), imaging and microbiological components. It reflects the presence of *Pseudomonas aeruginosa* colonization as an important predictive factor for poor prognosis, further contributing to the risk of hospitalization and mortality [12]. The FACED score entails the combination of FEV_1_, age, *Pseudomonas aeruginosa* colonization, radiological extension and dyspnea in predicting mortality; with the presence of *Pseudomonas aeruginosa* colonization associated with higher mortality [13]. Among these different predictive factors, *Pseudomonas aeruginosa* colonization could be a preventable or modifiable factor. As such, if factors that determine or contribute to *Pseudomonas aeruginosa* colonization could be identified, this may be an opportunity to intervene so as to reduce the chance of *Pseudomonas aeruginosa* colonization, and hence, to reduce the risk of hospitalization or mortality in non-CF bronchiectasis subjects. To date, the reported factors of *Pseudomonas aeruginosa* colonization are related to the severity of bronchiectasis, which are high sputum output, moderately severe airflow obstruction (FEV_1_/FVC < 60%) [2], older age (> 55 years), use of hypertonic saline for sputum induction, use of inhalation antibiotics, presence of primary ciliary dyskinesia and post-infectious etiology[14]. Whether these reported factors represent risk factors associated with *Pseudomonas aeruginosa* colonization or they are the consequences of *Pseudomonas aeruginosa* colonization or they predict *Pseudomonas aeruginosa* colonization is doubtful. This study aimed to assess the risk factors for *Pseudomonas aeruginosa* colonization in patients with non-CF bronchiectasis as well as its clinical and prognostic implications.

## Materials and methods

**Fig. 1:**
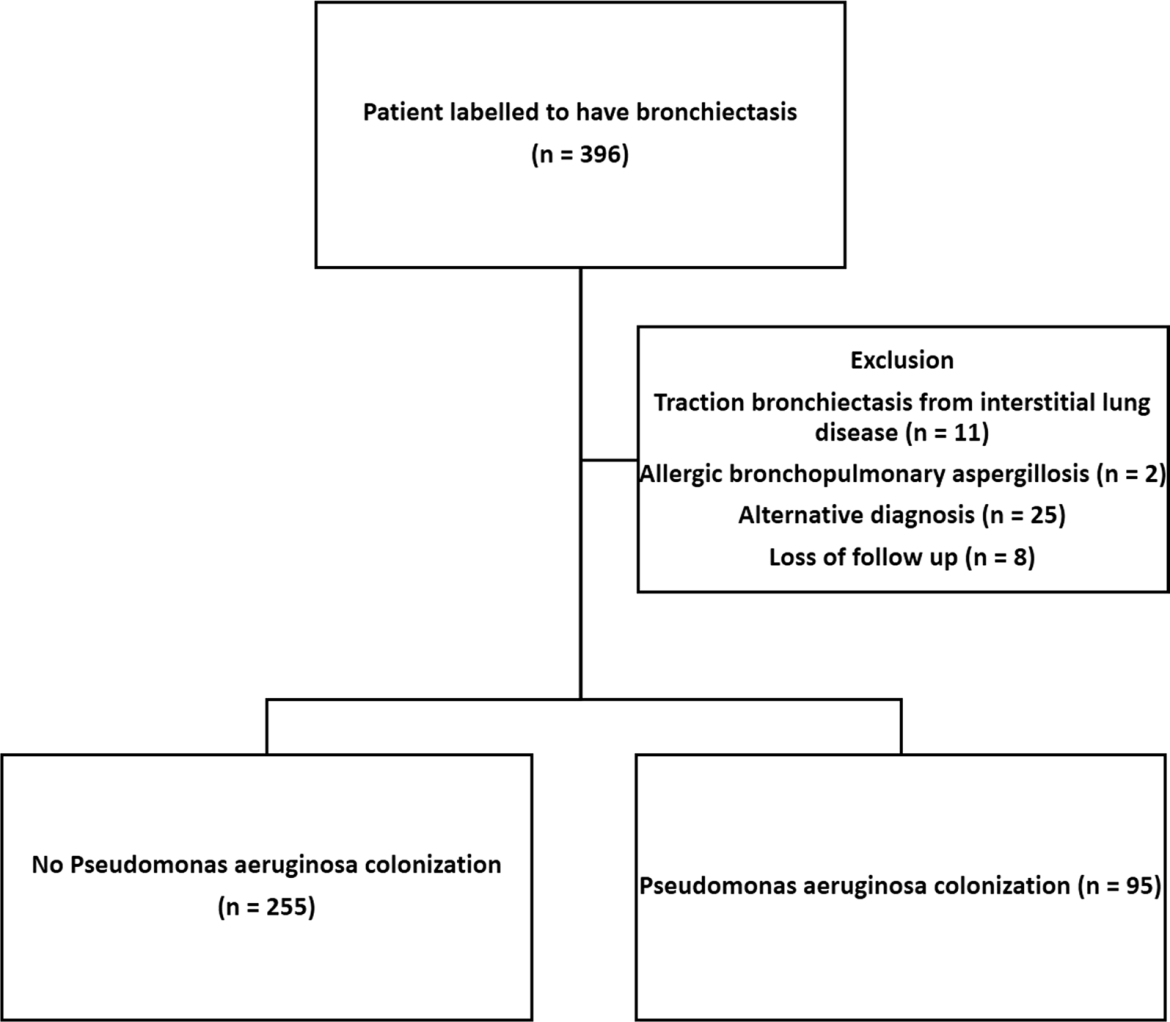
Flowchart of patient selection

This was a cross-sectional cohort study. All patients who have regular follow-up for non-CF bronchiectasis in the Respiratory Medicine Specialty Clinic in Queen Mary Hospital, Hong Kong in year 2018 were included. Our centre has a designated bronchiectasis clinic that follow up and manage patients diagnosed of bronchiectasis of different disease severity. The investigators reviewed the records and imaging findings to validate the diagnosis of bronchiectasis. Patients’ records were accessed through the electronic patient record (ePR) of the Hong Kong Hospital Authority, which consisted of the records of all patients with out-patient clinic attendances or hospital admissions. The information available includes demographics, clinical notes, investigation results and treatments. Patients’ information was further validated by the authors by reviewing the clinic record, relevant imaging, laboratory results and lung function test results. Exclusion criteria included traction bronchiectasis from interstitial lung disease, allergic bronchopulmonary aspergillosis, loss to follow up, alternative diagnosis and age less than 18 years old. Demographic data (age, gender, smoking status), clinical data / investigations (etiology of bronchiectasis, comorbidities, treatment records, spirometry results, sputum culture results), and use of PPI with indications were identified from clinical records. The patient selection process is summarized in Fig. 1.

Regular use of long acting beta-agonists (LABA), inhaled corticosteroids (ICS) or long-term macrolide were defined as continuous use of the medication for at least 12 months before recruitment into this study. PPI users were defined as those subjects who have been on regular PPI for at least 12 months before recruitment into this study and PPI use was continued after *Pseudomonas aeruginosa* colonization was identified. In our centre, patients with *Pseudomonas aeruginosa* colonization and repeated exacerbations were considered for home nebulized antibiotics or regular courses of intravenous antibiotics. For patients on regular intravenous antibiotics regime, elective admissions for anti-pseudomonal antibiotics were arranged every 12 to 16 weeks, with the choice of antibiotics based on the sensitivity pattern of the *Pseudomonas aeruginosa* isolates.

The primary outcome measure was the number or proportion of non-CF bronchiectasis subjects who have *Pseudomonas aeruginosa* colonization. The secondary outcome measures were the clinico-demographic factors associated with *Pseudomonas aeruginosa* colonization. *Pseudomonas aeruginosa* colonization was defined as the persistence of *Pseudomonas aeruginosa* in repeated sputum specimens or bronchoalveolar lavage taken at stable state without clinical evidence of infection and tissue damage within two years before recruitment [15]. Bronchiectasis exacerbation was defined as (1) a deterioration in three or more of the following key symptoms for at least 48 h, including cough, sputum volume and/or consistency, sputum purulence, dyspnea and/or exercise tolerance, fatigue and/or malaise, hemoptysis AND (2) clinician assessment that a change in bronchiectasis treatment was required usually with administration of antibiotics treatment [16]. Patients who had bronchiectasis exacerbations that required in-patient care from 1/1/2019 to 31/12/2019 were identified from the ePR of the Hong Kong Hospital Authority. The study was approved by the Institutional Review Board of the University of Hong Kong and Hospital Authority Hong Kong West Cluster (UW 20-435) (Table 1).

**Table 1:** Baseline demographic and clinical characteristics of included patients

	No *Pseudomonas aeruginosa* colonization (n = 255)	*Pseudomonas aeruginosa colonization* (n = 95)	*P* values
Age (years), mean ± SD	67.6 ± 13	68.5 ± 11.9	0.595
Age range (years)	20–98	25–88	0.595
Male	107 (30.5%)	26 (27.4%)	0.012*
Smoking status			0.056
Ever-smoker	68 (26.7%)	16 (16.8%)	
Non-smoker	187 (73.3%)	79 (83.2%)	
Daily sputum volume (mL)	6.24 ± 13.5	17.4 ± 20.4	< 0.001*
FEV_1_ (L)	1.84 ± 0.62	1.40 ± 0.57	< 0.001*
FVC (L)	2.75 ± 1.12	2.18 ± 0.7	< 0.001*
Extent of involvement ≥ 3 lobes	86 (33.7%)	63 (66.3%)	< 0.001*
All exacerbations	39 (15.3%)	44 (46.3%)	< 0.001*
Exacerbations requiring hospitalization	16 (6.3%)	23 (24.2%)	< 0.001*
Co-existing respiratory conditions			
Tuberculosis	48 (18.8%)	24 (25.2%)	0.185
NTM colonization	28 (11.0%)	18 (18.9%)	0.050
Asthma	24 (9.4%)	11 (11.6%)	0.357
COPD	20 (7.8%)	6 (6.3%)	0.687
Treatment			
Long term macrolide use	9 (3.5%)	17 (17.9%)	< 0.001*
Inhaled corticosteroid use	58 (22.7%)	50 (52.6%)	< 0.001*
Regular intravenous antibiotics administration	0 (0%)	9 (9.5%)	< 0.001*
Nebulized antibiotics	1 (0.40%)	2 (2.10%)	0.056

*SD* standard deviation, *mL* milliliter; *Statistically significant; *FEV*_*1*_ forced expiratory volume in one second, *FVC* forced vital capacity, *NTM* non-tuberculous mycobacterium, *COPD* chronic obstructive pulmonary disease

### Statistical analysis

The demographic and clinical data were described in actual frequency or mean ± SD. Baseline demographic and clinical data were compared between the two groups (with or without *Pseudomonas aeruginosa* colonization) with independent *t*-tests. To identify the risk factors and outcomes of *Pseudomonas aeruginosa* colonization, univariate logistic regression analyses were performed. Multiple logistic regression modeling was used to assess for potential confounders. Known risk factors for *Pseudomonas aeruginosa* colonization—age, sputum volume and FEV_1_/FVC ratio were adjusted in multivariate logistic regression for risk factors of *Pseudomonas aeruginosa* colonization, and known poor prognostic factors within FACED scores–FEV_1_, age, extent of involvement and dyspnea were adjusted for clinical and prognostic features in patients with *Pseudomonas aeruginosa* colonization. The statistical significance was determined at the level of *p* = 0.05. All the statistical analyses were performed using the 26th version of SPSS statistical package.

## Results

A total of 350 Chinese patients with non-CF bronchiectasis managed in Queen Mary Hospital (QMH) were included in this study.

### Baseline characteristics

The mean age was 67.9 ± 12.9 years. There were more females (62%) and never smokers (76%). Asthma and COPD were present in 26 (7.4%) and 35 (10%) of the patients respectively.

179 (51.1%) had oral flora from previous sputum culture samples. *Pseudomonas aeruginosa* was the commonest colonizer, occurring in 95/350 (27.1%) of the whole cohort, followed by *Hemophilus influenza* (n = 42; 12%), *Klebsiella pneumonia* (n = 15; 4.3%), *Staphylococcus aureus* (n = 13; 3.7%), *Moraxella catarrhalis* (n = 4; 1.1%) and other micro-organisms (n = 2; 0.6%). 46 (13.1%) had non-tuberculous mycobacteria (NTM) colonization. There were nine on regular intravenous antibiotics. For those who presented before the age of 18 years, from 18 to 35, 36 to 50, and 51 to 65: 1/16 (6.3%), 2/29 (6.9%), 3/48 (6.3%), and 3/129 (2.3%) required regular intravenous antibiotics. For patients who presented after the age of 65 years (n = 125), none of them required regular intravenous antibiotics.

In our cohort, there was no patient having chronic lung infection with *Pseudomonas aeruginosa*, which was defined as the presence of *Pseudomonas aeruginosa* in the bronchial tree for at least six months, with at least three positive cultures separated by at least one month interval in-between them with clinical evidence of infection and tissue damage [15].

The results are summarized in Table 2. The antibiotics sensitivity pattern of patients with *Pseudomonas aeruginosa* colonization.

**Table 2:** Antibiotic sensitivity pattern of *Pseudomonas aeruginosa* recorded in this cohort of patients

Antibiotics resistance	Number (% total number of subjects)
No resistance (sensitive to all antibiotics)	67 (70.5%)
Anti-pseudomonal beta-lactam	1 (1.1%)
Anti-pseudomonal cephalosporin	1 (1.1%)
Fluoroquinolone	12 (12.6%)
Carbapenem	3 (3.2%)
Both anti-pseudomonal beta-lactam and anti-pseudomonal cephalosporin	3 (3.2%)
Both anti-pseudomonal beta-lactam and carbapenem	1 (1.1%)
Anti-pseudomonal beta-lactam, fluoroquinolone and aminoglycoside	1 (1.1%)
Both fluoroquinolone and aminoglycoside	2 (2.1%)
Both fluoroquinolone and carbapenem	2 (2.1%)
Fluoroquinolone, aminoglycoside and carbapenem	1 (1.1%)
Resistant to more than 3 classes of antibiotics	1 (1.1%)

### Risks of *Pseudomonas aeruginosa* colonization in patients with non-CF bronchiectasis

In univariate logistic regression, female gender, longer duration of bronchiectasis, proton pump inhibitor usage was associated with *Pseudomonas aeruginosa* colonization. After adjustment for age, sputum volume, FEV_1_/FVC ratio, extent of lung involvement, frequency of exacerbation and prior use of anti-pseudomonal antibiotics within one year of isolation of *Pseudomonas aeruginosa*, which were reported to be associated with *Pseudomonas aeruginosa* colonization, longer duration of bronchiectasis and PPI usage remained significantly associated with *Pseudomonas aeruginosa* colonization, with ORs of 1.028 (95% CI = 1.013–1.042, *p* < 0.001) and 2.144 (95% CI = 1.198–3.835, *p* = 0.010) respectively.

The results are summarized in Table 3.

**Table 3:** Risk factors for *Pseudomonas aeruginosa* colonization

Risk factors for *Pseudomonas aeruginosa* colonization	Univariate analysis Odd ratios and 95% CI	*p* values	Multivariate analysis	*p* values
Female	1.919 (1.146–3.211)	0.013		
Longer duration of bronchiectasis*	1.077 (1.053–1.102)	< 0.001	1.028 (1.013–1.042)	< 0.001
PPI use*	2.340 (1.326–4.127)	0.003	2.144 (1.198–3.835)	0.010

*Factors that were statistically significant after adjustment for age, sputum volume, FEV_1_/FVC ratio, extent of lung involvement, frequency of exacerbations and prior use of anti-pseudomonal antibiotics within one year of isolation of *Pseudomonas aeruginosa*

### Clinical features in patients with *Pseudomonas aeruginosa* colonization

In univariate logistic regression, patients with Pseudomonas aeruginosa colonization was shown to have higher sputum volume, more likely to have involvement more than 3 lobes of lung, more likely to experience dyspnea, have lower FEV_1_ in percentage predicted values, lower FVC in percentage predicted values, lower FEV_1_/FVC ratio, higher risk of exacerbations requiring hospitalizations.

In multivariate logistic regression, extent of involvement and also exacerbation requiring hospitalization remained statistically significant, with ORs of 2.445 (95% CI = 1.283–4.657, *p* = 0.007) and 2.745 (95% CI = 1.012–7.449, *p* = 0.047) respectively, after adjusted for the known poor prognostic factors within FACED score. The results are summarized in Table 4.

**Table 4:** Clinical features of patients with *Pseudomonas aeruginosa* colonization

Clinical features of patients with *Pseudomonas aeruginosa* colonization	Univariate analysis Odd ratios and 95% CI	*p* values	Multivariate analysis	*p* value
Sputum volume	1.041 (1.024–1.058)	< 0.001		
More than 3 lobes of lung involved*	3.869 (2.350–6.369)	0.001	2.445 (1.283–4.657)	0.007
Dyspnoea	2.673 (1.525–4.685)	0.001		
Lower FEV_1_	1.034 (1.020–1.047)	0.001		
Lower FVC	1.031 (1.016–1.047)	< 0.001		
Lower FEV_1_/FVC	1.031 (1.010–1.053)	0.004		
Risk of all exacerbations	4.778 (2.818–8.103)	< 0.001		
Risk of exacerbations requiring hospitalizations*	4.772 (2.393–9.516)	< 0.001	2.745 (1.012–7.449)	0.047

^*^Factors that are statistically significant after adjustment for FACED score components—FEV_1_, age, extent of involvement and dyspnea

## Discussion

In this single center study, *Pseudomonas aeruginosa* was the commonest bacteria colonizing the airways of patients with non-CF bronchiectasis. Our study identified that, other than previously reported risk factors, longer duration of bronchiectasis and PPI use were independent risk factors for *Pseudomonas aeruginosa* colonization, which have important clinical implications. While advanced age is a known poor prognostic factor, those who had longer duration of bronchiectasis had disease onset at younger age. These patients did not only have symptoms for longer duration and worse quality of life, they were also at increased risk of *Pseudomonas aeruginosa* colonization, which was associated with worse prognosis in bronchiectasis. Patients have *Pseudomonas aeruginosa* colonization at young age, it would be important to consider *Pseudomonas aeruginosa* eradication once it is isolated in sputum, as once *Pseudomonas aeruginosa* colonized the airway, the chance of successful eradication will be low. The European Respiratory Society (ERS) guidelines suggested eradication of new *Pseudomonas aeruginosa* infection [17]. Eradication of *Pseudomonas aeruginosa* can prevent the consequences from *Pseudomonas aeruginosa* colonization, which are greater decline in lung function and more frequent exacerbations, especially in younger patients. [17] It would also be essential to monitor and follow up these patients more closely and regularly.

PPI was another risk factor for *Pseudomonas aeruginosa* colonization identified in this study. In our cohort, there were total 67 patients on long term PPI. 37 (55.2%) were on PPI for primary prophylaxis, such as given with aspirin or non-steroidal inflammatory drugs; 19 (28.4%) were on PPI for gastro-esophageal reflux disease and 11 (16.4%) were on PPI for secondary prophylaxis after an episode of gastric ulcer. Previous studies suggested that PPI usage was associated with bronchiectasis exacerbation requiring hospitalization.[18] The association between PPI and *Pseudomonas aeruginosa* colonization may be mediated through gastric acid suppression, as previous studies suggested the association between chronic achlorhydria and risks of community acquired pneumonia [19] and acute gastroenteritis. [20] Gastric acid suppression in patients with bronchiectasis may lead to chronic hypochlorhydria or achlorhydria, promoting the growth of colonization in the lower airway, and hence *Pseudomonas aeruginosa* colonization. Retrograde colonization of *Pseudomonas aeruginosa* in the oral cavity with subsequent aspiration to lower airways could contribute to *Pseudomonas aeruginosa* colonization in lower respiratory tract among patients with bronchiectasis. A potential preventive measures would be to try to lie in semirecumbent position during sleep to avoid aspirating saliva with *Pseudomonas aeruginosa* down to the lower respiratory tract during sleep. Both PPI and *Pseudomonas aeruginosa* colonization are independent risk factors for bronchiectasis exacerbation, their synergistic effects on bronchiectasis exacerbation should not be under-estimated. As most other risk factors for *Pseudomonas aeruginosa* colonization and bronchiectasis exacerbation are non-modifiable, avoiding PPI usage should be considered in patients with low risk of gastrointestinal bleeding, and switching to low dose PPI or histamine-2 receptor antagonist should be considered. From our result, more than 80% of the patients on PPI did not have absolute indication for PPI use and might be able to step down their PPI dose or even switch to histamine-2 receptor antagonist which have less propensity in causing achlorhydria. For patients with bronchiectasis, especially who have more severe disease, the indication and dose of PPI should be carefully reviewed and consider weaker gastric acid suppressant if not contraindicated. Shorter course of PPI or PPI use on as needed basis would be another possible strategy. This may have impact on the sputum microbiology in terms of *Pseudomonas aeruginosa* colonization, as well as bronchiectasis exacerbation risk. Apart from PPI use, aspiration as well as antibiotics resistance would also promote colonization of *Pseudomonas aeruginosa* in the respiratory tract. Antibiotics use would also lead to the emergence of antibiotics resistance. Appropriate use of antibiotics in patients with bronchiectasis would prevent *Pseudomonas aeruginosa* colonization as well as emergence of antibiotics resistance. While prescription of fluoroquinolone as contingent stock courses is frequently practised to avoid unnecessary hospitalization for bronchiectasis patients with mild exacerbations, patients should be well informed on the appropriate indications to use the antibiotics courses.

Our study also suggested that the *Pseudomonas aeruginosa* colonization was associated with more severe disease, with more extensive involvement and increased risk of exacerbation requiring hospitalization. [2, 12–14] Our results were also in line with previous findings in Hong Kong that *Pseudomonas aeruginosa* colonization was associated with higher sputum volume and worse airflow obstruction with lower FEV_1_/FVC ratio. [2] In one report by *Martinez-Garcia *et al*.*, use of hypertonic saline for sputum induction and use of inhalation antibiotics were reported to be risk factors of *Pseudomonas aeruginosa* colonization. While the authors in the study did not specify the indications for sputum induction and use of inhalation antibiotics for their patients, it is reasonable to surmise that the use of therapies may reflect that the patients had more severe bronchiectasis requiring such treatments. In our cohort, no patient was prescribed hypertonic saline for sputum induction and few patients were on inhalation antibiotics. This is because long-term home nebulization therapy is difficult for most patients in Hong Kong, partly due to living environment that is considered to be sub-optimal. On the other hand, we have more patients with regular intravenous antibiotics. Patients on regular intravenous antibiotics in our centre were those who had more severe disease with *Pseudomonas aeruginosa* colonization and repeated exacerbations despite other measures. From our finding and what has been reported in the literature, patients with *Pseudomonas aeruginosa* colonization deserve more aggressive treatment to preserve lung function and to prevent exacerbation.

There are a few limitations in our study. First, this study involved only a single centre. However, being a tertiary medical centre, the respiratory unit receives referrals from all other health care sources. Patients diagnosed with bronchiectasis were managed in a designated bronchiectasis clinic in our centre. Second, the lung function tests were done at different time for the patients within this cohort. Despite this, the results from our study is consistent with what was previously reported in the literature. Some of the patients did not have lung function test done. As such, leastwise deletion was adopted which can provide less biased estimates and conservative results. [21]The definition of *Pseudomonas aeruginosa* colonization was adopted from an European consensus statement on cystic fibrosis. [14] The criteria for defining lung colonization with *Pseudomonas aeruginosa* was the presence of *Pseudomonas aeruginosa* in bronchial tree without direct (inflammation, fever, etc.) or indirect (specific antibody response) evidence of infection and tissue damage. Chronic lung colonization with *Pseudomonas aeruginosa* was defined as the presence of *Pseudomonas aeruginosa* in the bronchial tree for at least six months, based on at least three positive cultures separated with at least one month intervals in-between without direct (inflammation, fever etc.) or indirect (specific antibody response) evidence of infection and tissue damage. In a retrospective study setting, this definition was difficult to observe as the timing of respiratory tract specimen sampling was highly variable. As a retrospective study, the frequency and number of sputum specimens saved as well as pulmonary function tests and imaging were not standardized. This may lead to a small degree of under-estimation on the number of patients with *Pseudomonas aeruginosa* colonization in this cohort. In order to understand the clinical course of patients with non-cystic fibrosis bronchiectasis, a well-designed study with long duration of follow up will be worth-while.

## Conclusion

*Pseudomonas aeruginosa* colonization is more common among patients with longer duration of bronchiectasis and those on PPI. *Pseudomonas aeruginosa* colonization is associated with more extensive lung involvement and higher risks of exacerbation requiring hospitalization, despite more intense regular treatment for bronchiectasis.

## Data Availability

Nil.

## Abbreviations

BSI: Bronchiectasis Severity Index
ePR: Electronic patient record
FEV: Forced expiratory volume
FVC: Forced Vital Capacity
ICS: Inhaled corticosteroids
LABA: Long acting beta-agonists
MMRC: Modified Medical Research Council
Non-CF: Non-cystic fibrosis
NTM: Non-tuberculous mycobacteria
PPIs: Proton pump inhibitors
SPSS: Statistical Product and Service Solutions
QMH: Queen Mary Hospital
